# Age-associated alterations in immune and inflammatory responses in captive olive baboons (*Papio anubis*)

**DOI:** 10.3389/fragi.2024.1511370

**Published:** 2025-01-06

**Authors:** Michele M. Mulholland, Bharti P. Nehete, Ashley DeLise, Angela M. Achorn, Lisa M. Pytka, Pramod N. Nehete

**Affiliations:** ^1^ The University of Texas MD Anderson Cancer Center Bastrop, Department of Comparative Medicine TX, Bastrop, TX, United States; ^2^ The University of Texas Graduate School of Biomedical Sciences at Houston, Houston, TX, United States

**Keywords:** aging, baboon, cellular immune response, FLUROSPOT, elispot, proliferation, cytokines

## Abstract

**Introduction:**

Advanced age is a primary risk factor for many chronic diseases and conditions; however, age-related immune dysregulation is not well understood. Animal models, particularly those that resemble human age-related physiological changes, are needed to better understand immunosenescence and to improve health outcomes. Here, we explore the utility of the olive baboon (Papio anubis) in studying age-related changes to the immune system and understanding mechanisms of immunosenescence.

**Methods:**

We examined immune cell, inflammatory responses, cytokines, and cortisol levels using hematology and flow cytometry, mitogen stimulation, multiplex cytokine assay, and cortisol immunoassay.

**Results and Discussion:**

Our results reveal significant age effects on numerous immune and inflammatory responses. For instance, adult and aged monkeys exhibited significantly fewer monocytes than young monkeys. After stimulation with Con A and PWM (separately), we found that old baboons had higher INFγ expression compared to young baboons. Similarly, after stimulation with LPS and PWM (separately), we found that old baboons had higher TNFα expression compared to young baboons. These findings suggest that the olive baboon is a suitable model for biogerontology research, immune senescence, and development of vaccines. Though there are phenotypic and functional similarities between baboons and humans, specific differences exist in immune cell expression and immune function of lymphocytes that should be considered for better experimental outcomes in the development of therapeutics and restoring innate and adaptive immune function in aged individuals.

## Introduction

In most countries, the average life expectancy has increased over the last century, with current lifespans averaging 80–100 years (Beltrán-Sánchez et al., 2015). As a result of this increased lifespan, elderly individuals are at higher risk for infection and may experience long-lasting antigen burdens which can trigger chronic immune system activation (Weyand and Goronzy, 2016; Karagiannis et al., 2023; Haynes, 2020). To mitigate the effects of chronic stress and activation, the immune system is reshaped and remodeled–a process through which some parameters remain the same, while others may increase or decrease with age. Adaptive immunity is one phenomenon that is significantly affected by aging, characterized by expanded memory and effector T cell clones and is more prominent in CD8^+^ cells and is specific for viral antigens (Whiting et al., 2015; Ramasubramanian et al., 2022).

Advanced age is a primary risk factor for severe chronic diseases and conditions which is proving to be considerably challenging to our healthcare system as the human population is aging rapidly. However, dysregulated immunity related to aging is not well understood. Animal models, particularly those that recapitulate human age-related physiological changes, are needed to better understand immunosenescence and improve health span (Kirkland and Peterson, 2009). Non-human primates (NHPs) have played a critical role in biomedical science due to their phylogenetic closeness to humans in structure and function (Campos et al., 2021; Sapolsky and Altmann, 1991; Snyder-Mackler et al., 2020). Species, such as rhesus macaques and marmosets, have served as sophisticated translational models of aging and age-related diseases such as cancer, diabetes, arthritis, cardiovascular disease and neurological decline (Mattison and Vaughan, 2017; Phillips et al., 2014a; Abbott et al., 2003). There is rising demand for NHP use in biomedical research particularly for studies of the brain, disease (cancer, Alzheimer’s, Parkinson’s, diabetes/obesity, addiction), infectious disease (HIV/AIDS, Ebola, Zika), and birth outcomes (miscarriage, stillbirth, and premature birth) (Granoff et al., 1997; Perry et al., 2012; Gurung et al., 2018; Cai et al., 2004; Cox et al., 2013; Lizarraga et al., 2020).

Baboons serve as a nonhuman primate model of many human-related conditions and diseases. Olive baboons (*Papio anubis*), in particular, have served as models of diabetes, cardiac diseases, respiratory diseases, xenotransplantation, reproductive and neonatal physiology, as well as infectious disease, immunology, and vaccine development (McGill Jr et al., 1981; Wolf et al., 2006; Murthy et al., 2006; Magden et al., 2020; Ekser et al., 2010; Bailey et al., 1985; Heffernan et al., 1995; Chiu et al., 2013; Mahlke et al., 2021; Tarantal et al., 2022). Baboon are unique among NHPs in that, similar to humans, they have four classes of immunoglobulin G (IgG) and have comparable susceptibility to pathogens (Shearer et al., 1999). Additionally, a number of comorbidities exist among both aged baboons and humans including circadian rhythm sleep disorders, cognitive and memory impairments, loss of motor skills, cataracts and macular degeneration, diabetes, hypertension, amyloid deposition, osteoporosis, and atherosclerosis (LaCreuse and Herndon, 2003; Shively and Clarkson, 2009; Phillips et al., 2014b). In primates, prevalence of these diseases–particularly, atherosclerosis–increases with age and with consumption of a Western diet (Williams, 2011; Shively and Clarkson, 2009; Kelly et al., 2013). Of particular relevance here, Garcia-Vilanova et al. (2024) has found age-related differences in baboon immune responses to infectious disease (SARS-CoV-2). Their response to SARS-CoV-2 infection is similar to humans, with acute respiratory distress, prolonged viral RNA shedding, and increased lung inflammation, particularly in aged baboons (Garcia-Vilanova et al., 2024).

The baboon immune system shares many similarities with human immune systems, particularly with IgG subclasses 1, 2, 3, and 4, which make them an excellent model for vaccine development studies (Attanasio et al., 2002). Dysfunction of the immune system related to age leads to increased disease mortality in elderly individuals, due to both immunosenescence and excessive inflammatory responses (Sikora et al., 2021; McFarlane et al., 2011; Rivera-Hernandez et al., 2020). Therefore, an attractive mitigation to improving health and quality of life in elderly populations includes enhancement of immune function. Since baboons share physiological and immunological similarities to humans they serve as an excellent model for assessing complex aging processes. The goal of this study was to identify biomarkers of age-related changes to the innate and adaptive immune system phenotypically and functionally, using non-invasive measures of cytokines, chemokines, and cortisol as a step toward progressing research of immunosenescence and inflammation in animal populations. This will enable us better understand aging of immune cells and the function of immunity as a critical step to understanding differences in human aging.

## Methods

### Subjects

Subjects included 74 specific pathogen free olive baboons *(P. anubis);* including 64 females (30 dam-reared, 34 nursery-reared) and 10 males (5 dam-reared, 5 nursery-reared). All subjects were socially housed in indoor/outdoor runs or Primadomes™ at the Michale E. Keeling Center for Comparative Medicine and Research within The University of Texas MD Anderson Cancer Center, Bastrop, TX (UTMDACC).

All subjects were fed a high protein NHP diet (Lab Diet^®^ #5045), fresh produce, regular dietary enrichment (e.g., forage, seeds, nuts, peanut butter, frozen juice) and had *ad libitum* access to water. UTMDACC has a comprehensive behavioral management and veterinary program to assess baboon health and psychological wellbeing. All subjects included in the current study were healthy and free of illness. All blood samples were collected when the animals were already sedated for their biannual physical exams. All research was conducted according to the provisions of the Animal Welfare Act, PHS Animal Welfare Policy, and the principles of the NIH Guide for the Care and Use of Laboratory Animals and was approved by the Institutional Animal Care and Use Committee at the UTMDACC.

### Clinical and laboratory assessment of study animals

As in our previous work (Magden et al., 2020), the baboons were sedated for routine biannual physical exams and blood samples were collected. These samples were analyzed for a complete blood count (Siemens Advia 120 Hematology Analyzer, Tarrytown, NY) and serum chemistry profile (Beckman Coulter AU680^®^ Chemistry Immuno Analyzer, Brea, CA). The absolute number of lymphocytes, obtained from hematological analysis, was used in converting the frequency of the lymphocyte population obtained from the FACS analysis to get the absolute number of the lymphocyte subset populations.

### Flow cytometry

Fluorescence labeled monoclonal antibodies specific to different lymphocyte subsets was used to determine cell-surface markers from EDTA whole blood as described (Magden et al., 2020): B cells (CD20 APC clone L27, BD Pharmingen, San Jose, CA), T cells (CD3 PerCP, clone SP34-2, BD Pharmingen), NK cells (CD16 PE clone 3G8, BD Pharmingen, San Jose, CA), and monocytes (CD14 FITC clone M5E2 BioLegend, San Diego CA). Surface staining of whole blood for phenotype analysis of lymphocytes in peripheral blood was done as described previously (Magden et al., 2020). Using Celesta (BD Biosciences) flow cytometer, we were able to measure expression of T cells, B cells, NK cells, NKT cells, and monocyte populations along with their subsets from whole blood. We measured the expression of CD4, CD8 (CD3^+^CD4^−^) on lymphocytes and monocyte subsets. We defined NK cells as CD3^−^CD16^+^ and NKT cells as CD3^+^CD16^+^. Neutrophil population, defined as the CD66abce population, was selected from the granulocyte population (Supplementary Figure S1). These cell populations and subsets were gated on a forward scatter *versus* side scatter dot plot and analyzed using FlowJo software (Tree Star, Inc., Ashland, OR); see Supplementary Figure S1 for the flow gating strategy. Compensation and isotype controls were used to define specificity. All antibodies (see Table 1) used in this study were deemed cross reactive with the *P. anubis* species as previously reported (Magden et al., 2020) and from the NIH Nonhuman Primate Reagent Resource core facility (http.//www.NHPreagents.org). CBC hematology tests were performed to receive absolute values of the various immune cell types studied for further analysis.

**TABLE 1:** List of Human monoclonal antibodies used for analysis.

	Antibody	Color	Clone	Catalog #	Isotype	Company
1	CD3	APC-Cy7	SP34.2	557,757	Mouse IgG1,λ	BD Pharmingen
2	CD4	PerCP	L200	550,631	Mouse IgG1k	BD Pharmingen
3	CD14	FITC	M5E2	555,397	Mouse IgG2ak	BD Pharmingen
4	CD16	BV650	3G8	563,692	Mouse IgG1k	BD Pharmingen
5	CD20	APC	L27	340,941	Mouse IgG1	BD
6	CD66abce	PE	TET2	130-117-699	Mouse IgG2b	Miltenyi

### Multiplex cytokine analysis

Using plasma collected from different age-sex groups of *P. anubis*, we performed the multiplex cytokines assay using non-human primate cytokine/chemokine growth factor Panel A magnetic bead panel. Briefly, a 96-well filter plate was blocked with assay buffer at room temperature for 10 min, washed, and 25 µL of standards or controls were added to the appropriate wells. Then 25 µL of beads were added to each well and the plate was incubated on a plate shaker overnight at 4°C. The next day, after washing the plate two times with wash buffer, the plate was incubated for 1 h at room temperature with the respective detection antibody. Following this incubation, 25 µL of Streptavidin-Phycoerythrin was added for 30 min at room temperature. Then, the plate was washed two times with wash buffer and 150 µL of sheath fluid was added for reading. All steps of incubations were performed on a shaker. Multi-analyte profiling was performed on the Bio-Plex 200 system (Luminex X MAP technology). Calibration microspheres for classification and reporter readings as well as sheath fluid, assay and wash buffer were also purchased from Millipore Corporation. The acquired fluorescence data was analyzed by the Bio-Plex manager 5.0. The minimum detectable concentration in pg/mL for the following analytes, IFN- γ (1.37), IL-2 (0.59), IL-8 (0.02), IL-1b (0.18), TNF- α (4.19), TNF-β (0.20), IL-6 (0.41), IL1RA (1.04), IL-21 (1.33), IL-4 (3.27), IL-5 (0.75), IL-10 (0.37), and IL-22 (2.09), was calculated by the Multiplex Analyst immunoassay analysis software from Millipore (Shelton et al., 2019; Magden et al., 2020).

### ELISPOT assay for detecting mitogen-specific INFγ and TNFα producing cells

Peripheral Blood Mononuclear Cells (PBMCs) isolated from fresh EDTA whole blood were stimulated with Con A, LPS, and PWM as previously described (Magden et al., 2020). To determine the number of INFγ and TNFα producing cells in a FluoroSpot ELISPOT assay we modified the methodology reported prior (Magden et al., 2020; Gurung et al., 2018). Briefly, PBMCs were aliquoted into duplicate wells (105/well) of a 96-well plate (polyvinylidene difluoride backed plates, MAIP S 45, Millipore, Bedford, MA) pre-coated with INFγ and TNFα antibodies. The plates were then incubated at 37°C for 36 h before the cells were removed, the plate was thoroughly washed with PBS, and developed as per the protocol provided by the manufacturer. Spots were produced representing individual cells secreting INFγ or TNFα counted by the Mabtech IRIS (Mabtech, Cincinnati, OH, United States of America). Samples were considered positive when the number of spots forming cells (SFCs) of the test antigen were at least five above the background control values from cells cultured in medium alone.

### Free cortisol measurement in baboon plasma by ELISA

A direct ELISA for plasma cortisol was carried out in a standard 96-well microtiter plate according to the protocol described in the Salivary Cortisol Enzyme Immunoassay Kit (Salimetrics, Philadelphia, PA, United States of America). This is a competitive immunoassay kit; cortisol in standards and samples compete with cortisol conjugated to horseradish peroxidase for the antibody binding sites on a microtiter plate. Plasma samples frozen at −80°C were thawed at 4°C and clarified by centrifugation and diluted 1:50 in assay buffer provided in kit. 25 μL of diluted plasma was used in duplicate for free cortisol measurement. After incubation, unbound components were washed away. Bound cortisol enzyme conjugate was measured by the reaction of the horseradish peroxidase enzyme to the substrate tetramethylbenzidine (TMB). This reaction produces a blue color, and after stopping the reaction with an acidic solution the color changes to yellow. The optical density is read on a standard plate reader at 450 nm. The amount of cortisol enzyme conjugate detected is inversely proportional to the amount of cortisol present in the sample.

### Statistical analyses

All analyses were performed with IBM SPSS Statistics (Version 26) and figures were created in GraphPad Prism (Version 10). Prior to analysis, outliers were removed from the dataset based on boxplots of each dependent variable. Three age groups were created: young (2–5 years old; 18 females, 5 males), adult (6–11 years old; 23 females, 2 males) and old (12–22 years old; 23 females, 3 males). Multivariate analyses of covariance (controlling for sex) were used to examine age and rearing differences in immune cells and pro-inflammatory markers followed by *post hoc* comparisons with Bonferroni corrections. Finally, bivariate correlations were run to examine the relationships between age and all variables. For all analyses, significance was tested at the *p* < 0.05 level.

## Results

### Hematology and flow cytometry

We previously described the flow cytometry panel designed to quantitate the relative frequency of major PBMC subsets in the specific pathogen free (SPF) and conventional baboon colonies housed at MD Anderson (Magden et al., 2020) and to examine potential differences as a function of Zika infection (Gurung et al., 2018). We first analyzed whole blood to find out the relative frequency of major PBMC subsets, i.e., T, CD4^+^ and CD8^+^, B, monocytes, and NK cells. Then we utilized a modified panel to examine age-related differences in lymphocyte subsets. Two outliers were removed; therefore, 72 baboons were included in these analyses.

**FIGURE 1:**
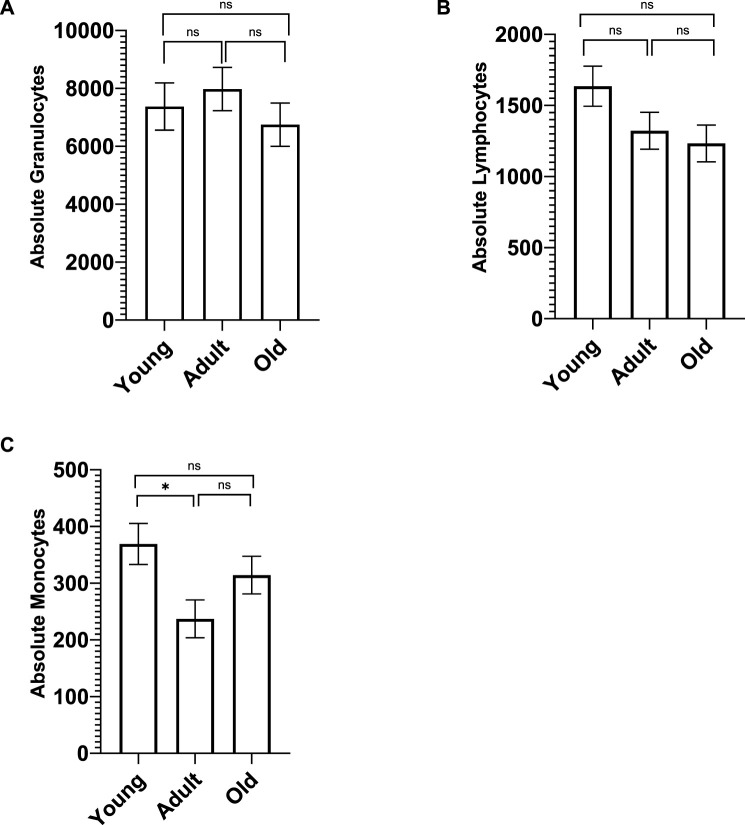
Whole blood chemistry was analyzed on Advia (Siemens Advia 120 Hematology Analyzer, Tarrytown, NY). There were no significant age differences in the number of granulocytes **(A)** and lymphocytes **(B)**. However, young monkeys had significantly more monocytes **(C)** compared to adult monkeys, but not old monkeys. Bars represent means and s.e.m., * indicates *p* < 0.05.

A MANCOVA (controlling for sex) found significant differences in white blood cell subtypes (granulocytes, lymphocytes, and monocytes) across age; *F* (6,128) = 2.473 *p* = 0.027 *η* ^*2*^ = 0.104. Specifically, monocytes differed [*F* (2,65) = 3.635 *p* = 0.032 *η* ^*2*^ = 0.016] with young monkeys having significantly more monocytes (*M* = 369.117 *SE* = 36.240) compared to adult monkeys (*M* = 237.018 *SE* = 33.305; *p* = 0.029) but not the old monkeys (*M* = 314.174 *SE* = 33.194; *p* > 0.05). There was no main effect of rearing or age by rearing interaction (*p* > 0.05). There were no significant age differences in granulocytes or lymphocytes (see Figure 1).

**FIGURE 2:**
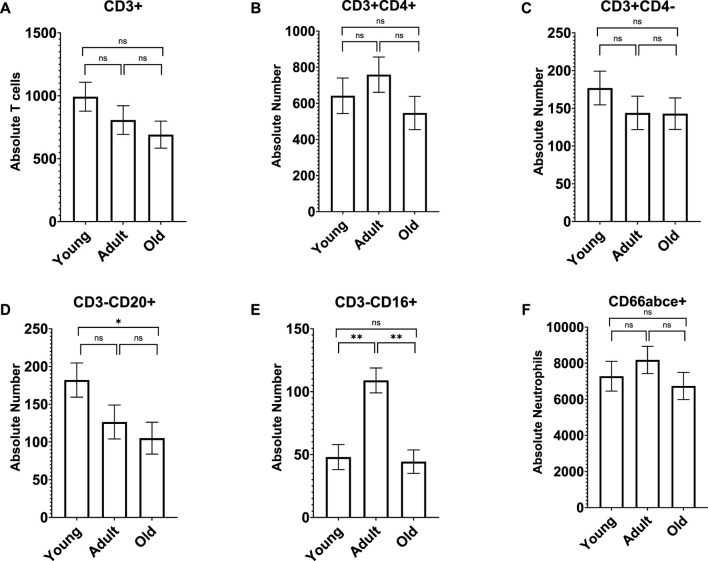
Old monkeys had significantly fewer CD3^−^CD20^+^ cells **(D)** than young monkeys but not adults. In addition, adult monkeys had significantly more CD3^−^CD16^+^ cells **(E)** than both young and old monkeys. There were no other age differences in lymphocyte subtypes **(A–C)** or neutrophils **(F)**. Bars represent means and s.e.m., * indicates *p* < 0.05, ** indicates *p* < 0.01.

A MANCOVA (controlling for sex) found an overall significant effect of age on neutrophils and lymphocyte subsets; *F* (12,122) = 3.655 *p* < 0.001 *η* ^*2*^ = 0.283. Both CD3^−^CD20^+^ and CD3^−^CD16^+^ differed across the three age groups [*F* (2,65) = 3.206 *p* = 0.047 *η* ^*2*^ = 0.090 and *F* (2,65) = 13.717 *p* < 0.001 *η* ^*2*^ = 0.297, respectively]. For CD3^−^CD20^+^, old monkeys (*M* = 105.038 *SE* = 21.154) had significantly fewer cells than young monkeys (*M* = 182.039 *SE* = 22.652; *p* = 0.047) but not adults (*M* = 126.465 *SE* = 22.455; *p* > 0.05). For CD3^−^CD16^+^, adult monkeys had significantly more cells (*M* = 108.927 *SE* = 9.886) than both young (*M* = 47.978 *SE* = 9.973; *p* < 0.001) and old monkeys (*M* = 44.362 *SE* = 9.313; *p* < 0.001); see Figure 2. There was no main effect of rearing or age by rearing interaction (*p* > 0.05). A MANCOVA (controlling for sex) found no significant differences in monocyte subtypes across age or rearing groups (*p* > 0.05; see Supplementary Figure S2).

**FIGURE 3:**
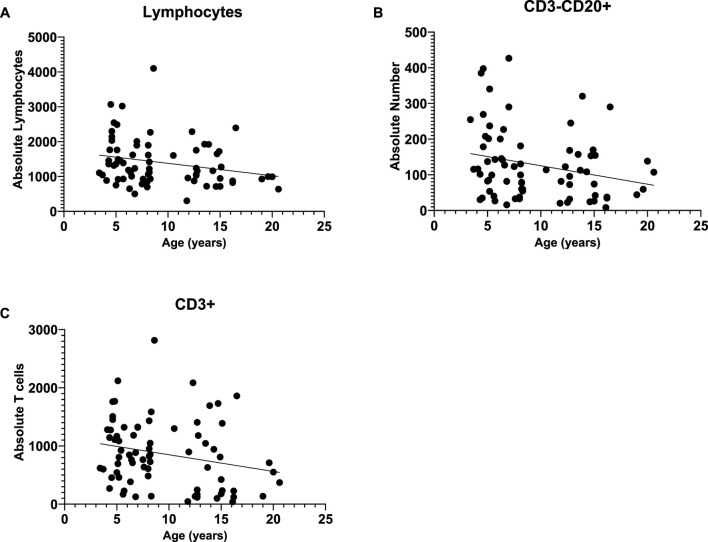
We found significant negative linear relationships between age and absolute lymphocytes **(A)** and CD3^−^CD20^+^ cells **(B)**, with both decreasing as baboons increase in age. Further, there was a marginal negative linear relationship between age and CD3^+^ **(C)**.

Since there were no rearing effects and sex was not a significant covariate in any of the above analyses, we combined data across rearing groups and sexes, then ran bivariate correlations to examine the linear relationship between age (years) and immune cell measures. We found significant linear relationships between age and absolute lymphocytes [*r* (73) = -0.247 *p* = 0.035] and CD3^−^CD20^+^ [*r* (73) = -0.272 *p* = 0.020], with both decreasing as baboons increase in age (see Figure 2). Further, there was a marginal negative linear relationships between age and both CD3^+^ [*r* (74) = -0.228 *p* = 0.051]; see Figure 3 for scatterplots. There were no other significant linear relationships (*p* > 0.05).

### Cytokines

Cytokine data was available for all 74 baboons; however, not all cytokines for each individual were above the detectable limit. MANCOVAs (controlling for sex) found no main effect of age, rearing, and no age by rearing interaction on the Th1 cytokines (*p* > 0.05), Th2 cytokines (*p* > 0.05), nor cytokines that fall under multiple categories (*p* > 0.05). See Table 2 for sample sizes and descriptive statistics.

**TABLE 2:** Means (SEM) of plasma cytokine levels across the age groups (adjusted for sex). Sample sizes indicated for the cytokine types for each age group.

Cytokine	Young	Adult	Old
Th1	n = 22	n = 18	n = 26
IFGγ	20.26 (6.21)	1.22 (6.85)	3.97 (5.73)
IL-2	10.70 (4.05)	3.09 (4.46)	4.91 (3.74)
IL-8	62.35 (9.73)	37.53 (10.73)	45.55 (8.98)
IL-1b	1.29 (0.33)	0.23 (0.36)	0.44 (0.30)
TNFα	103.33 (35.05)	3.33 (38.66)	8.70 (32.35)
TNFβ	0.53 (0.38)	0.74 (0.42)	0.79 (0.35)

Th2	n = 20	n = 4	n = 13
IL-6	5.65 (1.70)	6.91 (3.81)	2.62 (2.29)
IL-1RA	1.51 (0.34)	0.30 (0.77)	0.55 (0.46)
IL-21	0.32 (0.16)	0.37 (0.35)	0.26 (0.21)
IL-4	11.01 (4.02)	1.35 (9.01)	2.32 (5.41)
IL-5	5.09 (1.88)	1.64 (4.21)	1.49 (2.53)

Th1/Th2	n = 22	n = 18	n = 26
IL-10	2.77 (0.96)	2.21 (1.06)	1.30 (0.88)
IL-22	1.30 (0.53)	2.49 (0.58)	1.71 (0.49)

### Mitogen-specific expression of INFγ and TNFα

To examine age-related changes in cytokines secreted by cells, we collected whole blood from young, adult, and old baboons and isolated PBMCs using the Ficoll-Paque method (Magden et al., 2020) and responses to specific mitogens were measured by IFNγ, and TNFα via Fluorospot assay. Due to the limited PBMCs available after previous analyses, data from 64 baboons were included in the analyses of the pro-inflammatory markers.

**FIGURE 4:**
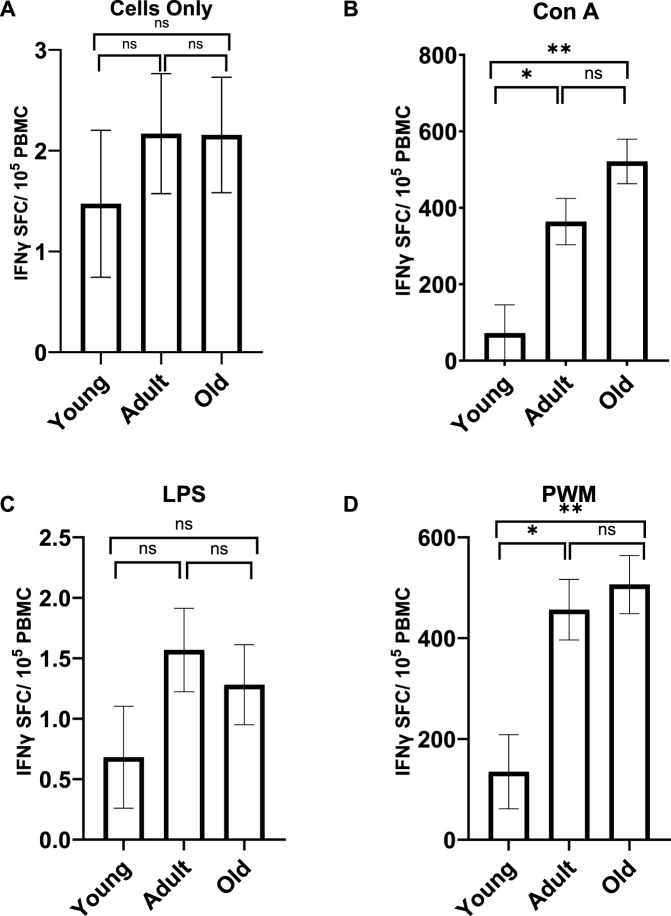
INFγ expression with no stimulation **(A)** or after LPS stimulation **(C)** did not differ across the age groups. However, the age groups did differ in INFγ expression after Con A **(B)** and PWM **(D)** stimulation. Young monkeys showed significantly lower INFγ expression compared to both adult and old monkeys. Bars represent means and s.e.m., * indicates *p* < 0.05, ** indicates *p* < 0.01.

A MANCOVA (controlling for sex) found a significant effect of age on INFγ; *F(*8,92) = 3.359 *p* = 0.002 *η* ^*2*^ = 0.226. Both Con A and PWM stimulations resulted in proinflammatory marker differences across the three age groups [*F* (2,48) = 11.411 *p* < 0.001 *η* ^*2*^ = 0.322 and *F* (2,48) = 8.65 *p* = 0.001 *η* ^*2*^ = 0.265, respectively; see Figure 4]. For INFγ expression after Con A stimulation, young monkeys showed significantly fewer cells (*M* = 71.920 *SE* = 74.244) compared to both adult (*M* = 363.906 *SE* = 60.703; *p* = 0.012) and old monkeys (*M* = 521.350 *SE* = 58.304; *p* < 0.001). Similarly, for INFγ expression after PWM stimulation, young monkeys had significantly fewer cells (*M* = 135.014 *SE* = 73.439) compared to both adult (*M* = 456.510 *SE* = 60.046; *p* = 0.005) and old monkeys (*M* = 506.360 *SE* = 57.672; *p* = 0.001). There was no main effect of rearing or age by rearing interaction (*p* > 0.05).

**FIGURE 5:**
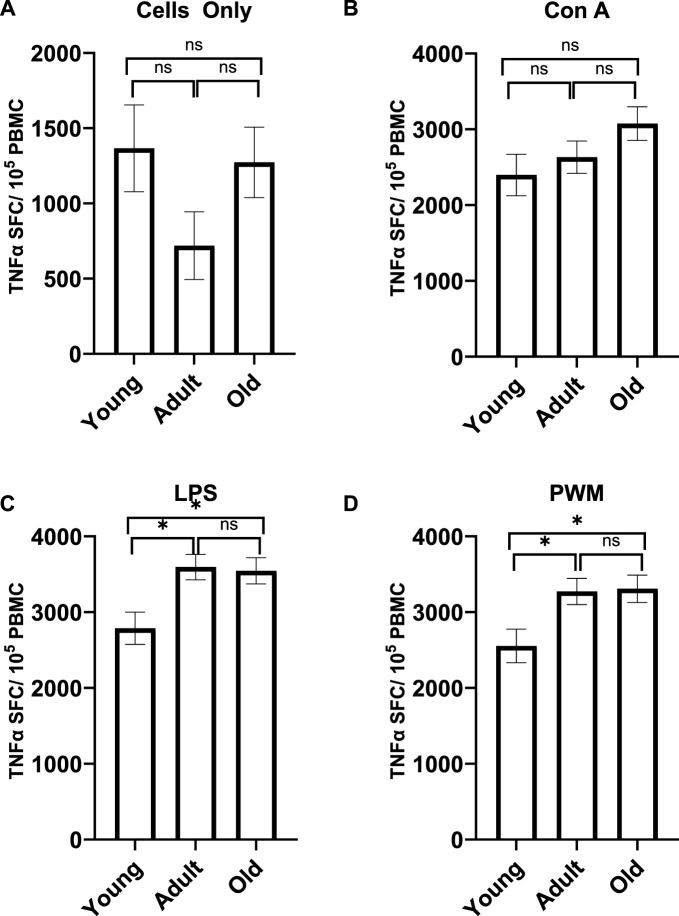
TNFα expression with no stimulation **(A)** or after Con A stimulation **(B)** did not differ across the age groups. However, the age groups did differ in TNFα expression after LPS **(C)** and PWM **(D)** stimulation. Young monkeys had significantly lower TNFα expression compared to both adult and old monkeys. Bars represent means and s.e.m., * indicates *p* < 0.05.

A MANCOVA (controlling for sex) found a significant effect of age on TNFα expression; *F* (8,110) = 2.729 *p* = 0.009 *η2* = 0.166. Differences across the three age groups were observed with LPS and PWM stimulations [*F* (2,57) = 5.134 *p* = 0.009 *η* ^*2*^ = 0.153 and *F* (2,57) = 4.174 *p* = 0.020 *η* ^*2*^ = 0.128, respectively; see Figure 5]. For TNFα expression with LPS stimulation, young monkeys had significantly fewer cells (*M* = 277.047 *SE* = 213.327) compared to both adult (*M* = 3594.241 *SE* = 166.164; *p* = 0.013) and old monkeys (*M* = 3544.801 *SE* = 173.16; *p* = 0.023). Similarly, for TNFα expression with PWM stimulation, young monkeys had significantly fewer cells (*M* = 2554.751 *SE* = 221.943) compared to both adult (*M* = 3273.046 *SE* = 172.875; *p* = 0.040) and old monkeys (*M* = 3308.365 *SE* = 180.139; *p* = 0.032). There was no main effect of rearing or age by rearing interaction (*p* > 0.05).

**FIGURE 6:**
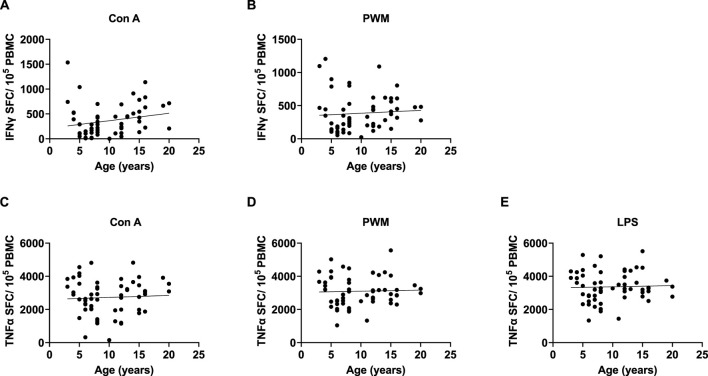
We found significant positive linear relationships between age and INFγ expression with Con A and PMW stimulation **(A and B)**, as well as TNFα expression with Con A, PWM, and LPS stimulation **(C–E)**.

Since there were no rearing effects and sex was not a significant covariate in any of the above analyses, we combined data across rearing groups and sexes, then ran bivariate correlations to examine the linear relationship between age (years) and each measure of these pro-inflammatory markers. There were significant positive linear relationships between age and INFγ expression with Con A stimulations [*r* (62) = 0.384 *p* = 0.002], INFγ expression with PMW stimulation [*r* (62) = 0.345 *p* = 0.005], TNFα expression with Con A stimulation [*r* (62) = 0.309 *p* = 0.013], TNFα expression with LPS stimulation [*r* (62) = 0.263 *p* = 0.036], and TNFα expression with PWM stimulation [*r* (62) = 0.309 *p* = 0.013]. See Figure 6 for scatterplots.

### Free cortisol

Cortisol data was available for all 74 baboons. An ANCOVA (controlling for sex) found no significant differences in cortisol across age groups, rearing groups, nor any age by rearing interactions (*p* > 0.05; see Supplementary Figure S3).

## Discussion

Overall, we found several age-related differences in immune and inflammatory responses in olive baboons. First, utilizing CBC hematology data, we found age differences in absolute monocytes but not in absolute lymphocytes, granulocytes, or neutrophils. For monocytes, young baboons had significantly greater monocyte counts compared to adult baboons, but did not differ from aged baboons, showing a decrease in numbers in adulthood and an increase in old age. Age differences in absolute monocytes have also been documented in marmosets (Hickmott et al., 2024; Mietsch et al., 2020) and cynomolgus monkeys (Xie et al., 2013). There are, however, conflicting findings for rhesus monkeys, with some studies reporting negative correlations between age and absolute monocytes (Didier et al., 2012) and others reporting no relationship (Smucny et al., 2001).

We used flow cytometry to further examine absolute lymphocytes and lymphocyte subsets. We found a significant negative relationship between age and absolute lymphocytes, with numbers decreasing with age, similar to what has been reported for marmosets (Hickmott et al., 2024) and some rhesus monkeys (Didier et al., 2012). When examining the lymphocyte subsets, we found that CD3^−^CD20^+^ B cells, and CD3^+^ T cells decreased with age, and when examining differences across age groups, old baboons had a lower number of CD3^−^CD20^+^ B cells compared to young baboons, and a lower number of CD3^−^CD16^+^ NK cells compared to adult baboons. Similar age differences in absolute B cells was found in Bolivian squirrel monkeys (Nehete et al., 2013). However, no such age differences in B and NK cells were found in chimpanzees or owl monkeys (Nehete et al., 2017a; Nehete et al., 2017b), and Chinese rhesus macaques showed the opposite relationship for CD3^+^ T cells, with percentages increasing with age (Qiu et al., 2008). We, and others, define NK cells as CD3^−^CD16^+^ and NKT cells as CD3^+^CD16^+^ cells (Magden et al., 2020; Kennett et al., 2010; Malyguine et al., 1996; Rappocciolo et al., 1992). There are several subsets of NK cells that do not express CD16, while small monocytes, which are CD3^−^may express CD16 (Forconi et al., 2020a; Forconi et al., 2020b). In baboons, less than 1% of lymphocytes express CD56, natural killer cell markers, compared to human peripheral blood lymphocytes (Malyguine et al., 1996). Recently, transcriptomic analysis of healthy cynomolgus macaques revealed important aging patterns with each gene cluster representing a different immune response predominantly associated with innate immune cells, such as neutrophils and NK cells, causing chronic inflammation and with adaptive immunity, especially “B cell activation” affecting antibody diversity of aging (Cho et al., 2024). Here we observed higher monocyte counts in young baboons and elevated NK cell counts in adult baboons that are inconsistent with those reported for other NHP models. In free-ranging rhesus macaques (Rosado et al., 2024), reported age-associated higher proportions of cytotoxic cells, NK cells, T regulatory cells, intermediate monocytes, and classic monocytes. Factors, other than age, may affect the composition of immune cells such as sex, social environment, social status, and habitat characteristics/living conditions (e.g., wild, free-ranging, captive, etc.), which may explain some of the inconsistences between our findings and those of other NHP models. For example, long-term studies of wild baboons in Amboseli suggests that social and early life factors can impact survival (Tung et al., 2023).

It is important to note that we observed some contradictory findings between the hematology assays and flow cytometry. Our hematology assays showed that young monkeys had significantly more monocytes than adult monkeys but in flow cytometry analysis no difference found in the number of CD14^+^ cells. In addition, we found no differences in the absolute number of lymphocytes via hematology, but a significant linear relationship with age with the flow cytometry results. These differences between flow cytometry and hematology may be due to flow cytometers displaying higher results and better performances than other methods (Buoro et al., 2018). The Society of Hematology and the International Council for Standardization in Hematology have both recently suggested that the use of a panel of monoclonal antibodies in flow cytometry may soon replace optical and digital microscopy as the reference technique (Hübl et al., 1995).

After stimulation with Con A and PWM (separately), we found that old baboons had higher INFγ expression compared to young baboons. Similarly, after stimulation with LPS and PWM (separately), we found that old baboons had higher TNFα expression compared to young baboons. In addition, we found significant correlations between age and both INFγ and TNFα expression, with expression of both proinflammatory markers increasing with age after stimulation with PMW, INFγ expression increasing with age after Con A stimulation, and TNFα expression increasing with age after LPS stimulation. Again, this differs from our previous work with chimpanzees, squirrel monkeys, or owl monkeys (Nehete et al., 2013; Nehete et al., 2017a; Nehete et al., 2017b).

Finally, we found no differences in cortisol or cytokines across the age groups. Again, this contrasts with what we have reported for owl and squirrel monkeys (Nehete et al., 2013; Nehete et al., 2017b) and others have reported for rhesus monkeys (Didier et al., 2012). Geriatric owl monkeys had significantly higher concentrations of IL-2, IL-12, INFγ, TNFα, IL-4, IL-6, IL-10, and IL-13 cytokines than both juveniles and adults (Nehete et al., 2017b). Similarly, in squirrel monkeys, aged individuals had significantly higher levels of IL-6, IL-10, IL-12, INFγ compared to both adult and young individuals, and significantly lower IL-2 compared to young individuals (Nehete et al., 2013). In rhesus monkeys, IL-6, IL-12, INFγ, and TNFα, all increased with age (Didier et al., 2012). Interestingly, McFarlane et al. (2011) found that when examining longitudinal changes in cytokines in captive baboons (in a sample likely related to our own), both IL-6 and IL-6/IL-10 ratio increased with age. It is that possible we would see similar relationships with repeated sampling in our subjects.

Baboons share genetic, physiological, and immunological similarities to humans and are susceptible to human pathogens, making them an important preclinical model for studies of pathogenesis and its causal mechanisms (Wilson et al., 2023; Mask et al., 2022; Colman, 2018; Slayden and Martin, 2018). The results here demonstrate the utility of the olive baboons (*P. anubis*) in studying age associated changes of the immune system. We observed significant decreases in monocytes, B cells, and NK cells in aged baboons. Similarly, we also found significant linear relationships between age and absolute lymphocytes, both decreasing as monkey age increased. These findings are consistent with clinical studies and point to the responsibility of adaptive immunity and T cell compartment changes in the decreased immunity with old age (Chaplin, 2010; DeMaio et al., 2022; Messaoudi et al., 2011).

The “immune risk phenotype” or the impacts of immunosenescence on both the innate and adaptive immune system is related to longevity (Wikby et al., 2005). We observed an increased INFγ and TNFα immune responses to mitogen stimulations in both adult and older baboons in comparison to the younger baboons. These findings suggest that while adult and aging baboons share some phenotypic and functional similarities, there are specific differences in immune cell expression or lymphocyte function, which may affect the quality and quantity of innate and adaptive immune responses (Magden et al., 2020; Bjornson-Hooper et al., 2022; Barreiro et al., 2010).

Because NHPs are genetically and physiologically similar to humans, they are naturally susceptible to a wide range of human pathogens and pathogens closely related to those that infect humans. This makes them an invaluable model for understanding disease pathogenesis, immune system function, and for vaccine or therapeutic development (Shively and Clarkson, 2009; Voytko et al., 2009). Baboons, in particular, have similar immune systems to humans and similar effects of aging on the immune system, including reduced production and function of B and T cells associated with poor clinical outcome (Ponnappan and Ponnappan, 2011; Haynes and Maue, 2009). Our study found that young monkeys exhibited significantly more monocytes than adults, which resembles what has been reported in humans as they age. For instance, Cao et al. (2022) found that older adults had a significantly higher percentage of total monocytes in PBMCs and in whole blood compared to young adults and middle-aged adults. Our study also found that after stimulation with Con A and PWM (separately), old baboons had higher INFγ expression compared to young baboons. This is different from what we see in humans; Murasko et al. (1986) report that elderly humans (70–90 years old) showed decreased mean responses to Con A compared to the young control subjects, and there was no significant difference in responses to PWM between elderly and young subjects. It is important to note here that the oldest baboon in our sample was 22, which corresponds to a human age of approximately mid-60s (Bredbenner et al., 2013). Therefore, it is possible that older baboons (30+ years old) would show similar decreases in mitogen responses as elderly humans. Lastly, we found no significant differences in cortisol across the age groups for the baboons. This differs from what we generally see in humans, which is higher basal cortisol levels later in life (Moffat et al., 2020; Nicolson et al., 1997). It is important to note here that these studies in humans examined salivary and urinary cortisol levels, which reflects a different time frame than the plasma levels we examined here. A study of rhesus monkeys found increases in plasma cortisol levels with age (Erwin et al., 2004), but again included older subjects (up to 40 years old) than the current study. Future research should include a wider age range of baboons.

One potential limitation is that the findings here may not be generalizable to wild baboon populations as it includes captive specific pathogen free baboons. Studies with wild and captive baboon populations show marked variation in microbiota architecture and resistance across habitats and lifeways (Tsukayama et al., 2018) and wild baboons have a richer microbial diversity (Joseph et al., 2018). Even within captive environments there can be differences in immune responses related to varied pathogen exposure. A study of conventional and specific pathogen free colonies at our institution showed that conventional baboons showed greater T-cell proliferation to stimulation with phytohemagglutinin or PWM, and higher IFNy producing cells after stimulation with Con A or PWM or with Toll-like ligands TLR3, TLR4, and TLR8 (Magden et al., 2020). Selecting the appropriate population of baboons for future immunology studies is important and researchers should consider if a more natural immune system development and exposure to pathogens is warranted (i.e., a conventional baboon model) or a more controlled, limited previous exposure to pathogens (i.e., a specific pathogen free model) is needed for the study.

A better understanding of the NHP immune system will enhance studies of immune function and its role in the protection against infectious diseases, and further the development of novel vaccines specific for the vulnerable aging population (Kennedy et al., 1997; Coe and Ershler, 2001). Identifying and validating biomarkers of immunosenescence in an NHP is crucial, since, to date, there is no test or marker for immune senescence onset other than age. An alternative method to determine biological age based on an epigenetic or biomarker-based clock was recently described in baboons (Weibel et al., 2024). In a study of wild baboons in Kenya, DNA methylation-based age calculations revealed that high-ranking males are predicted to be older than their true chronological ages, and that this imposes costs consistent with a ‘live fast, die young’ life-history strategy (Anderson et al., 2021). The development of a similar immune marker-based clock would likely be helpful in determining how various lifestyle and demographic factors and novel age rejuvenation interventions [such as vaccination, etc. (Martin et al., 2021; Agrawal and Weinberger, 2022)] impact immune aging.

Overall, NHPs are essential in furthering our understanding of the mechanisms underlying immunosenescence and developing novel vaccination strategies. This is particularly important for the aging population, as increased susceptibility to infections and long-lasting antigen exposure are major causes of morbidity and mortality in this vulnerable group. A better understanding of the cellular and molecular mechanisms of aging will increase opportunities to intervene in aging and age-related diseases.

## Data Availability

The raw data supporting the conclusions of this article will be made available by the authors, without undue reservation.
