# Supplementary figures and images for: Age-associated alterations in immune and inflammatory responses in captive olive baboons (Papio anubis)
Source: Front Aging. 2025 Jan 6;5:1511370. doi: 10.3389/fragi.2024.1511370 (PMC11743478; doi:10.3389/fragi.2024.1511370)

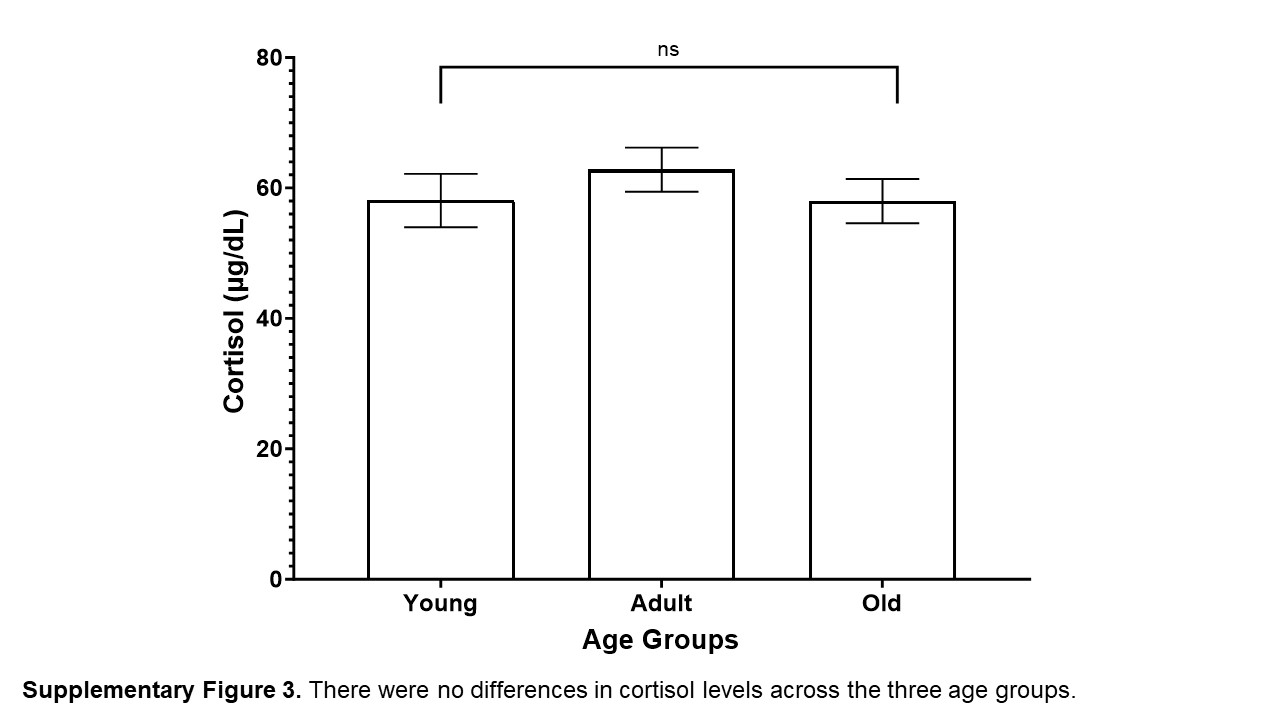

Supplement: Supplementary file 1 [file Image3.jpeg]

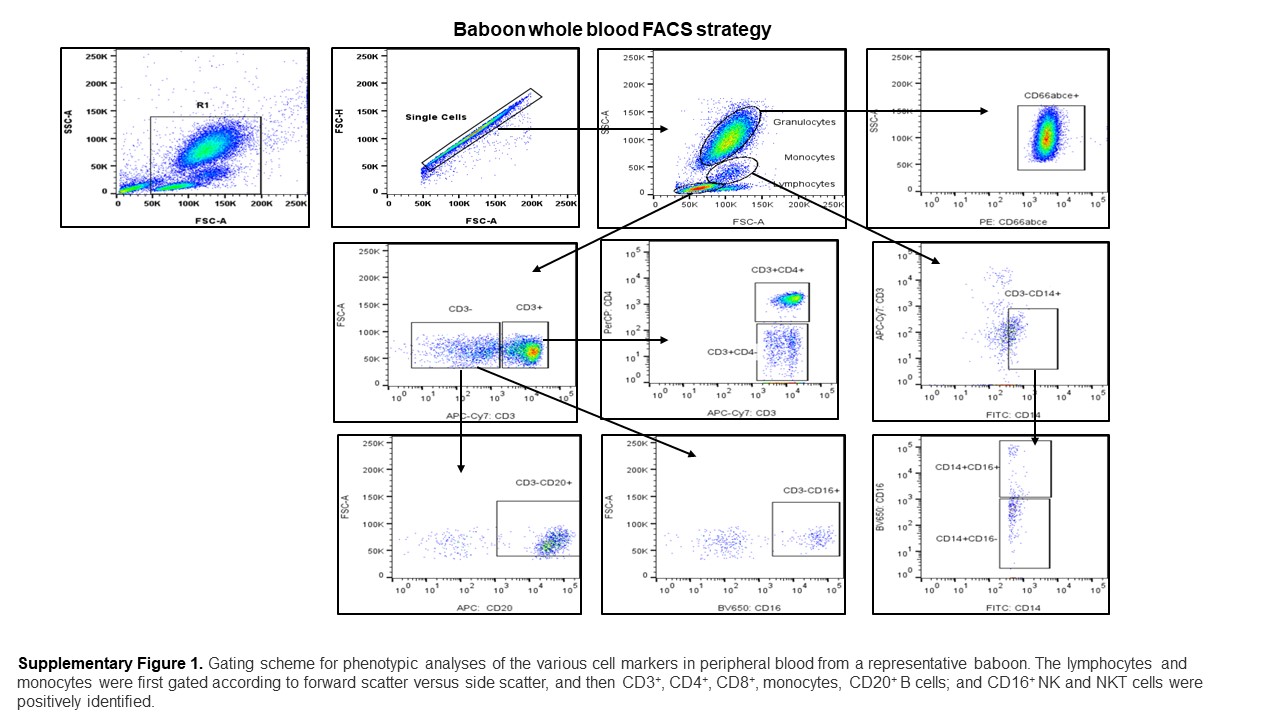

Supplement: Supplementary file 2 [file Image1.jpeg]

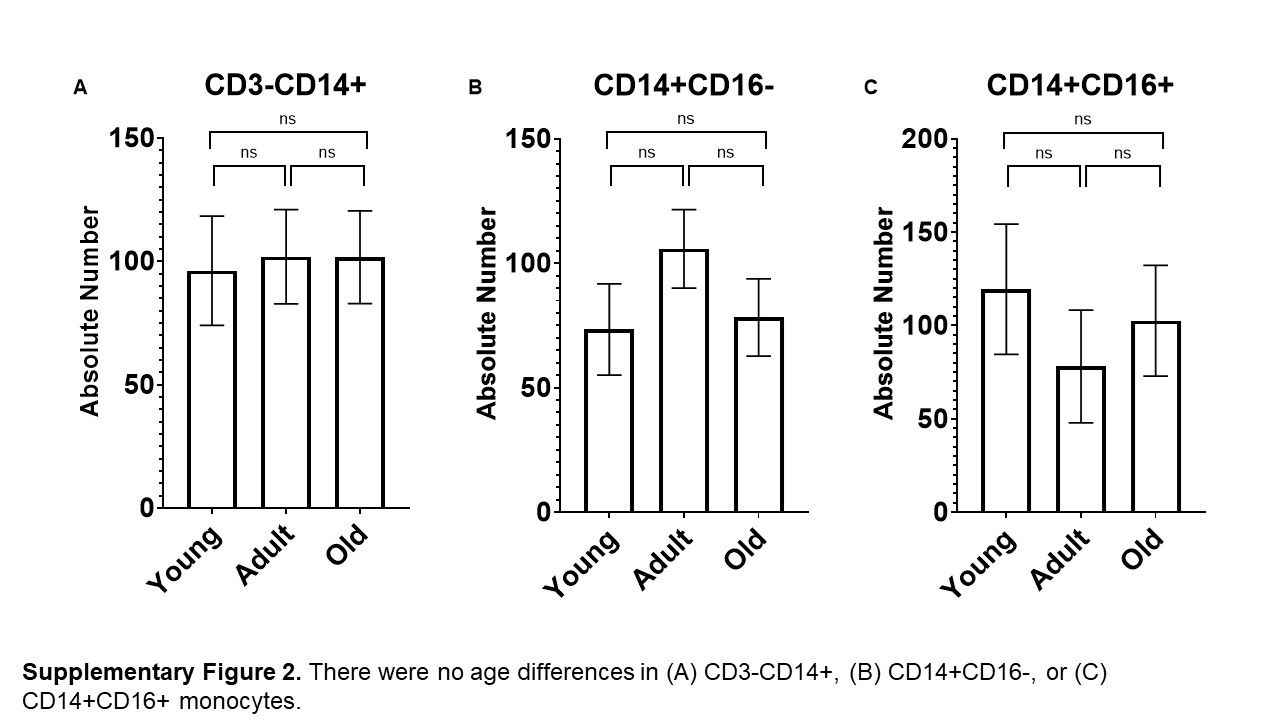

Supplement: Supplementary file 3 [file Image2.jpeg]
